# Pre‐Registration Mental Health Nursing Students Who Witness Self‐Harm Amongst Service Users During Placement: A Cross‐Sectional Study

**DOI:** 10.1111/inm.70200

**Published:** 2025-12-17

**Authors:** James Stockton, Steve Lui, John Stephenson, Maxine Cromar Hayes, David Solomon, Michael Haslam, Steve Hemingway

**Affiliations:** ^1^ Department of Social and Psychological Science, School of Human and Health Sciences University of Huddersfield Huddersfield UK; ^2^ Priory Hospital Leeds Leeds UK; ^3^ Department of Allied Health Professions, Sport and Exercise, School of Human and Health Sciences University of Huddersfield Huddersfield UK; ^4^ Institute of Population Health University of Liverpool Liverpool UK; ^5^ School of Nursing, Midwifery and Health Education Anglia Ruskin University Cambridge UK; ^6^ School of Nursing and Midwifery University of Central Lancashire Preston UK; ^7^ Department of Nursing and Midwifery, School of Human and Health Sciences University of Huddersfield Huddersfield UK

**Keywords:** higher education, mental health nursing students, placement, self‐harm, undergraduate

## Abstract

Mental health nursing (MHN) students may witness self‐harm on placements; however, little is known regarding the experience of MHN students who do witness this. This study aimed to understand the personal impact of witnessing self‐harm events upon MHN students who are on placement, with three objectives: (1) To identify the types of self‐harm witnessed by MHN students; (2) To evaluate MHN students' perceived self‐competence in working with service users who have self‐harmed; and (3) To assess the potential psychological trauma upon MHN students after witnessing self‐harm. A cross‐sectional questionnaire comprising researcher‐generated Likert‐style items and open‐ended response questions was utilised. Descriptive analysis was completed of 84 responses from MHN students. The types of self‐harm witnessed included cutting (65 participants; 77.4%); head‐banging/punching (62 participants; 73.8%); and ligation (36 participants; 42.9%). Additionally, we identified factors that either hindered or aided the MHN students in developing resilience post witnessing self‐harm. These findings are presented as three qualitative themes, and are: resilience; sources of stress and sources of support. Key findings were: (i) current MHN students can experience a range of negative outcomes after witnessing a self‐harm incident which can include wanting to withdraw from studies; (ii) student experience of witnessing self‐harm needs to be better understood and responded to; and finally, (iii) universities and placement providers should deliver relevant training, with trauma informed care (TIC) pedagogy potentially being an effective intervention. Ultimately, we recommend a joined‐up approach from universities and practice partners to address these issues.

## 1 Introduction

### 1.1 Background/Literature

Self‐harm has been defined as any form of self‐inflicted injury or poisoning and is common in inpatient mental health services (National Institute for Health and Care Excellence 2022). During a study in 2012, James, Stewart, and Bowers (2012); James, Stewart, Wright, and Bowers (2012) noted that 17.4% of mental health inpatients self‐harm within the ward setting. We submitted a freedom of information to the Mental Health Services Dataset, which showed that in 2024 a total of 78 786 self‐harm incidents in psychiatric wards were reported; although a decrease from 2023/2, the figures from 2024 are still twice that of 2016. Suggesting that rates of inpatients self‐harm has only increased since James et al.'s publication.

Whilst rates of inpatient self‐harm are rising (Karakaya et al. 2023) found that only 50% of nurses utilise knowledge and skills to positively and effectively respond to these crisis situations; this illustrates a potential care gap in how nurses respond to suicidal ideation and self‐harm acts. Dickens and Hosie (2018) investigated nursing reactions to self‐harm and found that these can vary from proactive and therapeutic to reactive and coercive. The above information, in combination with pedagogical concerns from teaching experiences, served as key drivers in prompting the research.

In the United Kingdom, nursing students are required to complete 2300 h of placement work across the course of an undergraduate degree, providing opportunities to gain valuable and authentic experience (NMC 2018; Van der Riet et al. 2018). However, recent commentaries have questioned student experiences, arguing that not all students are prepared for MHN practice (Warrender 2022; Connell et al. 2022; Warrender et al. 2024), Just as placements can be positive learning environments, they can be uniquely challenging, with many students experiencing emotional and psychological struggles during placement (Davenport et al. 2018). In some cases, placements can be classified as failed, as students choose to withdraw from studies during or after the placement period. Contributing factors toward placement failure have been identified and include: marginalisation and lack of preparation (Younas et al. 2022), lack of contact time with Practice Supervisors/Assessors (Jacobsen et al. 2022), issues with personal mental health which can be exacerbated by undergraduate study (Tung et al. 2018; Clements et al. 2023), poor work life balance and financial struggles (Grant‐Smith and de Zwaan 2019).

Compounding the above, is that MHN students traditionally undertake placements that place them in highly stressful situations. These students are often exposed to traumatic situations that they may not have been given the tools or knowledge needed to overcome (Watson et al. 2020). These traumatic situations can include witnessing self‐harm. Witnessing this can be particularly problematic for students, as a lack of prior knowledge and skills can make encountering, responding to and recovering from a traumatic experience especially challenging (Galvin et al. 2015). Trauma here refers to recurrent negative and distressing outcomes following incident exposure; these include but are not limited to: recurring distressing thoughts whilst awake, persistent negative dreams of the incident, uncontrollable bodily reactions in response to reminders of the incident, difficulty falling asleep, concentration and/or increases in general anxiety. In recent years, TIC has emerged as a way to mitigate primary/secondary trauma of both patients and nurses. Although still in its infancy, TIC shows promise as a way of managing both patients and nurse trauma. This is of particular interest when considering the rates at which MHN students may be witnessing self‐harm and subsequently suffering from trauma (Watson et al. 2025; Wilson et al. 2024).

Making placements a more rewarding experience is a pressing issue, with the ongoing need for student nurses to graduate and join the nursing workforce. In the UK, the National Confidential Inquiry into Suicide and Safety in Mental Health (2024) has specifically recommended reducing staff turnover as a way of improving safety in acute settings, and preregistration experience is one area that needs to be targeted. In 2017, the UK had the third lowest proportion of currently practising nurses in the European Union, with only 2.8 nurses per 1000 individuals (Watson 2017). By 2021, there were a reported 50 000 nursing vacancies in the UK, and the turnover rate of nurses was increasing compared to previous years (Marufu et al. 2021; Kings Fund 2024). Newly graduated nurses often struggle in the transition period, and attrition rates of new nurses are high (See et al. 2023). Several issues pertaining to student attrition have been identified, including: personal inadequacy, feelings of vulnerability and personal transformation occurring after engaging in patient care (Soerensen et al. 2023). Similar issues to these have been echoed elsewhere, with the majority considering student nurse attrition rates to be the result of a long process of negative events and experiences, rather than a single issue. Ultimately, attrition through course withdrawal is the end of a decision‐making process that can span many years, and interventions aiming to reduce attrition should target multiple vectors (Watts et al. 2023; Solomon et al. 2024).

Ultimately, placements need to impart students with the knowledge and skill sets needed to be resilient to thrive in future employment. We define resilience as a MHN students' ability to both endure and successfully recover from witnessing a traumatic incident that is, self‐harm. An incoming MHN student lacking resilience is not at fault, and it should be the role of both universities and placement providers to foster an environment in which resilience can be developed in a safe and holistic manner. Zohn (2022) suggested that there is a need to proactively prepare nursing students for suicide prevention, which may help prepare student nurses for their crucial role in suicide prevention and negate potential negative issues (Croft et al. 2023). However, there is limited evidence on how this can be implemented, both in theory and in practice within the UK and worldwide (Ferguson et al. 2020).

Whilst placements are a demanding and challenging time in a course that is already replete with challenge, the experiences of MHN students who encounter self‐harm on placement are under‐researched. Because of this, opportunities may be missed to improve the student experience, and in turn, improve student retention and staff conversion (Heyman et al. 2015). The current study, which investigates the MHN student experience of self‐harm, adds knowledge to this important phenomenon and the preparation of MHN students for its likely occurrence.

### 1.2 Aim

To understand the personal impact of witnessing self‐harm upon MHN students whilst on placement.

### 1.3 Objectives

1. To identify the types of self‐harm witnessed by MHN students.
2. To evaluate MHN students' perceived self‐competence in working with service users who have self‐harmed.
3. To assess the potential trauma of witnessing self‐harm upon MHN students.

## 2 Methodology

### 2.1 Design and Data Collection

STROBE research design was followed, as this was a non‐longitudinal study (Von Elm et al. 2007). Data collection was completed using a cross‐sectional online survey tool via questionnaires including open‐ended and closed items. Recruitment to the study was via Mental Health Nurse Academic UK and involved four participating universities (University of Bedfordshire, University of Central Lancashire, University of Huddersfield, and University of Liverpool).

Data included participant demographic information including: course of study, age, gender, ethnicity, sexual orientation, previous experience in health/social care, previous experience in mental health care, previous personal experience of mental health, close friend or family member experience of mental health, previous experience of witnessing self‐harm in a clinical setting, instances of witnessing close friend or family member self‐harm, clinical setting and nation.

All types of self‐harm witnessed in high numbers were assessed for association with a previous history of self‐harm and the presence of connecting factors. Connecting factors included: failure to carry out observation level instructions; nursing staff allowing service users to leave hospital despite obvious risks; inadequate glazing on wards (i.e., non shatter‐proof glass installed); easy access to roofs or outside doors; unsupervised bathroom access; easy access to means of hanging; unsupervised access to lighters; failure to carry out adequate risk assessment; failure to prescribe appropriately; previous service user history of self‐harm.

Investigated separately, was how placement service staff had responded to promote service users' wellbeing following a witnessed incident of self‐harm. Placements were scored on the number of positive aspects featuring in their response including: evaluating triggers; observing signs of risk for self‐harm or self‐injury; implementing and providing prevention activities; allowing safe self‐harm under supervision; taking responsibility for patients' wounds and injuries; evaluating the need for medication; and additional nursing interventions. Participants were also asked about their immediate reaction to witnessing a self‐harm event. The strength of the effect of the incident on wellbeing was scored depending on the number of responses provoked, including wishing to discontinue a MHN course; feelings of incompetence or distress; feelings that leadership was unsupportive or staff made insensitive comments; avoiding conversations about the incident; and negative bodily reactions.

Qualitative information was collected via 6 open‐ended questions, relating to participant’ experiences on placement (questions shown in Table 1). Resulting information was analysed using inductive thematic analysis following the methods of Byrne (2021) and Graneheim and Lundman (2004). Whilst some participants did not respond to each qualitative question, across all participants and responses, we maintained a 90%+ response rate. Nineteen initial themes emerged, during bias/reliability discussion amongst authors these were reduced to 9 subthemes and finally complied into three distinct themes: *Resilience*; *Sources of Support*; and *Sources of Stress*. There were no pre‐established themes in place when analysing the dataset, instead responses were grouped by linguistic similarity, during grouping, we found some commonality in participant responses post witnessing service users self‐harm, which was reports of wanting to better understand how to support the service user in future, however not all participants reported this. Analysis did not show any single specific trait/outcome/belief that would encourage this positive response and so we theorised that participants who possessed greater levels of resiliency (defined in paragraph six of the introduction) in the wake of witnessing self‐harm events were more likely to seek positive outcomes post witnessing a self‐harm incident.

**TABLE 1:** Qualitative questions.

Question 1	What do you think is the general attitude of staff working in mental health services toward service users that engage in self‐harm/self‐injury?
Question 2	What has been the emotional impact upon yourself after working with service users who commit self‐harm/self‐injury?
Question 3	What are your views of your placement, in managing self‐harm/self‐injury behaviour?
Question 4	After witnessing an instance of self‐harm/self‐injury, did you feel supported, and what if any support was received?
Question 5	What style of support would you have liked to receive from placement providers after witnessing a self‐harm/self‐injury incident?
Question 6	As a mental health nursing student, what support would you have liked from the university after witnessing a self‐harm/self‐injury incident?

Using resilience as the primary theme, further analysis found positive and negative external factors that supported/hindered resilience, we classified these as sources of support/stress respectively. Sources of support involved such elements as: positive feelings toward placement/MHN, optimism post‐witnessing self‐harm, and perception of support from universities and placements. Whilst sources of stress included such elements as: poor self‐perception/worth, feelings of guilt/responsibility following witnessing self‐harm, and poor perception of placement/staff attitudes. For a full account of themes and subthemes, please see Table 2.

**TABLE 2:** Themes.

	Theme 1	Theme 2	Theme 3
	Resilience	Sources of support	Sources of stress
Subthemes	Resilience developed through exposure & experience	SHSI incidents handled positively by staff	Students perceive high levels of burnout and emotional fatigue amongst staff
	Post‐witnessing a SHSI incident, students want training to be better prepared for future incidents	Students felt supported by staff after witnessing SHSI	Students internalise feelings of guilt and shame after being involved in SHSI incidents
		Students receive, benefit from and desire debriefs post SHSI incidents	Students do not always feel included as part of a nursing team
			Students have concerns about the treatment of patients, especially those that have EUPD

## 3 Results

### 3.1 Quantitative Responses

The full descriptive demographic summary is found in Table 3, in brief, the current participants were predominantly bachelor students with clinical placement experience in the NHS and a history of health and social care experience. Across all participants, a total of 622 self‐harm events were witnessed throughout the entirety of their nursing experience: 22 participants (27.8%) witnessed 10 or more incidents, and 10 participants (12.6%) witnessed 20 or more incidents. The types of self‐harm incidents witnessed are presented in Table 4.

**TABLE 3:** Descriptive summary of sample.

Variable	Frequency (valid %)
Course of study (*n* = 83)	
Bachelor's degree	64 (77.1%)
Master's degree	19 (22.9%)
Gender (*n* = 80)	
Male	19 (23.8%)
Female	61 (76.3%)
Ethnicity (*n* = 83)	
White	43 (51.8%)
Black	32 (38.6%)
Other	8 (9.3%)
Sexual orientation (*n* = 83)	
Heterosexual	69 (83.1%)
Lesbian/gay/bisexual	13 (15.7%)
Other	1 (1.2%)
Health/social care experience (*n* = 83)	
Previous experience	59 (71.1%)
No previous experience	24 (28.9%)
Mental health experience (*n* = 83)	
Previous experience	43 (51.8%)
No previous experience	35 (42.2%)
Prefer not to say	5 (6.0%)
Mental health experience of personal family/friend (*n* = 83)	
Previous experience	57 (68.7%)
No previous experience	24 (28.9%)
Prefer not to say	2 (2.4%)
Experience of SHSI (*n* = 83)	
Previous experience	24 (28.9%)
No previous experience	55 (66.3%)
Prefer not to say	4 (4.8%)
Experience of personal family/friend SHSI (*n* = 83)	
Previous experience	35 (42.2%)
No previous experience	45 (54.2%)
Prefer not to say	3 (3.6%)
Clinical setting when witnessing SHSI (*n* = 83)	
NHS	58 (69.9%)
Private hospital	14 (16.9%)
Private practice	6 (7.2%)
Third sector/social enterprise	2 (2.4%)
Other	3 (3.6%)
Nation (*n* = 83)	
England	78 (94.0%)
Scotland	4 (4.8%)
Wales	1 (1.2%)

Variable	Mean (SD; range)
Age (years)	36.4 (10.1; 19–57)
Length (years) of previous health/social care experience	8.35 (6.00; 1–26)
Number of SHIS events witnessed (*n* = 79)	7.87 (10.6; 0–50)

**TABLE 4:** Type of SHSI witnessed.

Type of SHSI	Frequency (valid %)
Cutting	65 (77.4%)
Headbanging/punching/kicking	62 (73.8%)
Strangulation/suffocation/drowning	36 (42.9%)
Insertion of foreign objects into the body	24 (28.6%)
Reopening/interfering with pre‐existing wounds	36 (42.9%)
Burning	15 (17.9%)
Self‐poisoning	9 (10.7%)
Jumping through windows	3 (3.6%)
Jumping off roofs	1 (1.2%)
Jumping into moving traffic	3 (3.6%)
Any other method	9 (10.7%)

Higher incidence of witnessing self‐harm was reported by those with previous personal, friend, or family mental health experience and those who had themselves engaged in self‐harm or had a friend or family member who had done so. Commonly witnessed incidents involving cutting (77.4%), head‐banging (73.8%), strangulation (42.9%) and re‐opening of wounds (42.9%). At the time of witnessing the self‐harm event, service users were either under general observation (32.1%), intermittent observation levels (27.4%) or were kept within eyesight at all times (19%). Given the data, it seems that as observation levels decrease, self‐harm incidents increase; additionally, as previous history of witnessing self‐harm was the most common connecting factor associated with witnessing subsequent acts of self‐harm, it may suggest that experience increases awareness. Failure to carry out adequate risk assessment was a common connecting factor associated with incidents of cutting, head‐banging and re‐opening of wounds. Easy access to unsupervised bathrooms was a common connecting factor associated with incidents of cutting, strangulation and re‐opening of wounds. Easy access to a means of hanging was a common connecting factor associated with incidents of strangulation and head‐banging; which could refer to some form of solid beam that could be used when damaging the head. The number of incidents witnessed, by type of personal and family previous experience, is summarised in Table 5.

**TABLE 5:** Number of SHSI witnessed, by experience.

Variable	Incidents witnessed (mean (SD))
Health/social care experience (*n* = 79)	
Previous experience	6.02 (6.60)
No previous experience	12.1 (15.8)
Mental health experience (*n* = 79)	
Previous experience	9.27 (11.1)
No previous experience	5.89 (9.70)
Prefer not to say	11.3 (10.6)
Mental health experience of personal family/friend (*n* = 83)	
Previous experience	9.04 (11.7)
No previous experience	5.17 (6.93)
Prefer not to say	6.00 (n/a)
Experience of SHSI (*n* = 83)	
Previous experience	10.4 (13.3)
No previous experience	6.67 (9.24)
Prefer not to say	8.67 (5.51)
Experience of personal family/friend SHSI (*n* = 83)	
Previous experience	10.4 (12.2)
No previous experience	6.02 (9.13)
Prefer not to say	5.67 (4.51)

The number of connecting factors (maximum 7) observed by participants following a self‐harm incident, the corresponding placement response score (maximum 7) and the immediate resulting effect on the respondent are summarised in Table 6. The most commonly reported component of the “effect score” above was failure to be supported by their placement provider after witnessing a self‐harm event (23 participants; 27.4%). Other components featuring in many responses included wanting to discontinue their course of study (35 participants; 41.7%), experiencing feelings of incompetence (39 participants; 46.4%), and experiencing feelings of unsupportive leadership (29 participants; 34.5%).

**TABLE 6:** Sequalae of witnessing of SHSI events.

Variable	Mean (SD; range)
Number of connecting factors observed	1.88 (1.09; 0–6)
SHIS placement response score	3.66 (1.78; 0–7)
Immediate effect score	1.71 (1.62; 0–5)

### 3.2 Qualitative Responses

Whilst prior quantitative analysis had shown that many participants considered withdrawing from their studies after witnessing a self‐harm event, the results of the qualitative analysis instead showed that some participants sought positive outcomes and wanted to be more pro‐active during future incidents. We therefore theorised that in the immediate aftermath of witnessing a self‐harm event there is a strong desire to cease studies; however, some students possess the ability to overcome this desire and instead seek positive development. We have called this resilience and discuss further in theme one below. We also found that participant resilience is buttressed by supportive environments but can be undermined by sources of stress. We have discussed these additional findings further in themes 2 and 3 respectively.

#### 3.2.1 Theme 1: Resilience

Our prior quantitative information has shown that MHN students on placement are highly likely to witness a self‐harm incident. In the current dataset, participants who had witnessed self‐harm whilst on placement reported that this was usually a traumatic experience, with much of their trauma attributed to a lack of formal preparation:

[witnessing self‐harm] can be quite shocking as you're not used to seeing someone do that to themselves.

I had never seen self‐harm prior to this.

I initially felt very shocked and uneasy as I had a lack of experience working with self‐harm.

These initial findings concerning shock and trauma were largely in line with expectations from the quantitative data, however whilst we were expecting participants to then report negative outcomes for example, wanting to end studies, many of our participants instead reported wanting to better understand and support the service user, better understand how to anticipate and address potential triggers leading up to and during the event and how best to actively manage the event if it occurred again:

I think it will be good to know what to do afterward to help service users.

Teach us how to dress self‐harm wounds and de‐escalation strategies.

[At university] I would like more discussion on [self‐harm] and how we can better help the service user.

From these qualitative findings then, it seemed that whilst there is trauma associated with witnessing a self‐harm event, some participants did desire positive growth after the incident. In analysing this theme, we theorised that it is resilience, that mitigates against avoidant behaviour and instead facilitates this desire for positive growth.

Subsequent themes would also highlight the role that external factors can play in influencing the development of resilience, which we argue can only occur through witnessing a traumatic event and subsequently experiencing positive growth. Issues that may hinder the development of resilience are explored in theme two, and these have been termed as sources of stress. Contrasting these are sources of support, which were found to promote resilience, and these will be explored in the third theme.

#### 3.2.2 Theme 2: Sources of Stress

Multiple sources of stress were identified which can serve as a barrier to resilience development. These should not be considered mental/physical stress but are instead unique actions that limit the participants' cultivation of resilience. Both internal and external sources of stress were identified. Internal sources refer to beliefs, attitudes and thoughts participants developed after being involved in a self‐harm incident. These served as barriers to the participants developing the resilience to progress/develop in their role as an MHN, for example:

When witnessing self‐harm, I feel like [I] have let the [service user] down.

[I] felt bad because I could not help stop [the service user].

[I experienced feelings of] fear, low self‐esteem and hopelessness.

These examples illustrate how following a self‐harm incident, participants attribute blame to themselves, resulting in guilt. Whilst feelings of guilt may prompt further development of skillset, self‐blaming can potentially have long‐term ramifications, as seen in Compson (2015).

In contrast, externally occurring stress came from the actions of others, that is, perception of staff as lacking compassion. In the findings, participants reported that staff could be uncompassionate toward self‐harm incidents, especially if the service user engaging in the act had a diagnosis of personality disorder, with many participants saying that practicing nurses could be harsh in their treatment of self‐harm:

Sometimes I feel like staff make the service user feel bad for self‐harming. There is a huge stigma [for personality disorder] which needs to change.

Most staff members view [self‐harm] as an inconvenience, rather than an opportunity to help.

Participants also reported that they felt upset when told that these self‐harm actions were examples of attention‐seeking behaviour, and that their treatment of these service users should be adjusted accordingly. However, participants attributed these responses toward current nursing staff struggling with overwork, compassion fatigue and training:

Staff are overwhelmed

Staff are overworked [and undertrained]

Poor staffing and agency staff with little training/insight making up the bulk of the staffing

Additionally, participants sometimes reported being spoken to in a negative manner when they raised concerns surrounding self‐harm. These instances were described as upsetting and undermining:

You often get told to get used to it [self‐harm]

Quite often I was referred to as gullible or too soft

I feel like there was no support for students, and the only comments made were … this is … what it is like, get used to it.

Within this theme of stress, we found multiple factors which may hinder the development of resilience. Based on the analysis, it seems that if internalised, and left unresolved, these experiences can have serious ramifications in the continued success of MHN nursing students. Countering these, are sources of support, which serve as protective factors against sources of stress, discussed in theme 3 below.

#### 3.2.3 Theme 3: Sources of Support

Sources of support can serve as protective factors, encouraging participants to learn from and better cope with trauma. Information from participants implies that both placement and university staff can serve as sources of support:

I have been fortunate to have been on placements where the team has been very supportive of myself and each other.

I was able to spend time with the ward manager and some of the nurses on the shift to debrief.

In the last quote, we see the participant specifically highlight the important role that debriefs have in supporting them following a self‐harm incident. This finding applied to most participants, as almost all participants highlighted the importance and benefit of meaningful debriefs, which often serve as the first point of support following a self‐harm incident. When debriefs had not been performed, students regretted the absence of the debrief:

I was never asked if I needed a debrief afterwards, which I feel I would have greatly benefited from

Whilst debriefs are the support wanted from the placement provider, participants also highlighted ways in which their university could have supported them. Most of these desired sources of support involved further in‐depth training, suggesting that participants want to be more involved in the treatment of service users who engage in self‐harm:

I think more in‐depth training is required

More lessons on how to emotionally deal with [self‐harm fallout] in a more professional manner

The university has to get students more prepared both mentally and physically, before going to placement, in order to be able to handle what they might see in placement

To summarise this theme, participants call for more support typically in the form of debriefs and additional supervision; suggesting that support already exists, but more can be done. Students rely on both their placements and universities to create supportive environments but expect different levels of support from both. On placements, students draw support from social bonds, and managerial oversight through debriefs and one‐to‐one meetings. From universities, students draw support from the teaching, and staff oversight. All these have been highlighted as positive contributing factors in the development of resilience. We also highlight that participants are actively wanting to engage with these resources and do benefit from them. Results from these three themes have been used during the development of our proposed theoretical model of resilience development, which can be found in the discussion section.

## 4 Discussion

The results of this study revealed several interesting and concerning results regarding the experience of MHN students across multiple universities; these are particularly important considering the need for our MHN students to successfully transition into clinical practice. In addition to our own findings, a freedom of information request also showed an over two times increase in self‐harm incidents between 2016 and 2024, with a total of 78 786 incidents on psychiatric wards in 2024. This increase mirrors the high level of self‐harm reported by participants. Amongst our participants, 622 self‐harm incidents were reported, and the most common types of self‐harm witnessed were cutting, headbanging, self‐ligature and reopening pre‐existing wounds. The amount of self‐harm witnessed initially seemed high; however, considering broader trends in wards, these figures may be broadly similar across student cohorts. The Safewards model, which was launched in 2013 (Bowers et al. 2015), has attempted to address this important clinical issue of a safer patient‐driven ward environment and the use of a compassionate approach. However, evidence of effectiveness in promoting the culture of acute wards and outcomes is limited, and clinical governance and process of the implementation and training has not been consistent in worldwide studies (Ward‐Stockham et al. 2022; Ghoorun et al. 2025). Our evidence also suggests that self‐harm continued to be an increasing issue, with negative effects upon participants that witness this.

**FIGURE 1:**
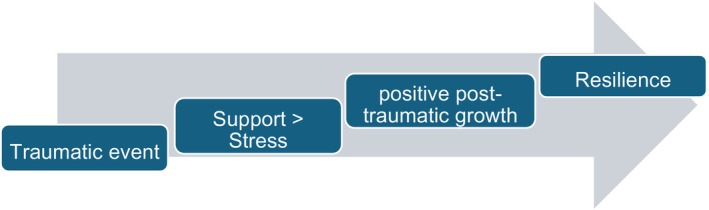
Theoretical staged model of resilience development.

Quantitative and qualitative analysis of this dataset has shown that almost 50% of participants had considered withdrawing from their course of study. In addition, most participants felt either distressed or incompetent after being exposed to self‐harm, with around 25% feeling that the placement was unsupportive. Given the need for retention, it is worrying that so many participants expressed a desire to withdraw from their course of study. A descriptive phenomenology by Zohn (2022) suggested that MHN students encounter a variety of challenges in adapting to their roles on placement, especially after witnessing a self‐harm incident; some of these challenges included avoiding burnout, struggling with the nursing role, and doubting their own skill sets. The current study draws similar conclusions to Zohn (2022); specifically, that the most impactful time in a MHN student's placement journey is the aftermath of a self‐harm incident and progression after the fact. Given that our results dovetail with findings from other papers, and that our results have also clearly identified factors that contribute to the hindering and development of resilience, we are tentatively proposing the below theoretical model of resilience development. This model has been developed primarily through analysis of the qualitative information. In particular, we looked at the interplay of trauma exposure and resilience development as mediated by sources of stress and support. As most of the qualitative information suggests that positive outcomes are a process rather than a guarantee, we have presented a model where support is greater than stress. If this was to be inverted, that is, stress greater than support, then we would expect resilience to decrease (Figure 1).

Within this model, we hypothesise that resilience to endure and overcome specific events follows a staged process. Firstly, the individual is exposed to the event; then if support outweighs stressors, positive growth can be achieved. Through repeated exposure and growth, resilience can be cultivated. In developing this model, we also integrated similar results from other authors who had explored resilience. Of these authors, Videbeck (2020) states that MHN students develop competence in managing suicide prevention on their courses in mental health nursing, which should provide fundamental knowledge regarding suicide, such as epidemiology, risk factors, signs or symptoms of suicide, and discussions about assessments and interventions. Zohn (2022) similarly found that the emotions and stress related to these challenges can affect students. Ko‐Sun et al. (2019) also reported that nursing students can feel frustrated and powerless in such situations, echoing these findings. Karakaya et al. (2022) stated that nurses' knowledge and skill in dealing with suicidal behaviour, including self‐harm, should include how to seek help and support post‐incident. The proposed model also integrates arguments of Ho et al. (2024) that resilience in the face of adversity is the result of a process of interactions rather than possession of a singular fixed trait. This model also sidesteps the attribution of blame/guilt on the part of MHN students who are deemed not resilient enough by making resilience something that is developed in tandem with others, rather than an intrinsic trait, which echoes arguments from Fisher and Jones (2024).

Whilst the model attempts to outline a process of resilience development, further work is needed in future to develop this more robustly; however, our current results should raise serious discussion as to the issues that mental health nursing students encounter whilst on placement, and the best ways to support them. Whilst existing literature has surveyed registered nursing experiences, our results strengthen this by exploring the experiences of MHN students (Kane et al. 2022; Clements et al. 2023). As the next generation of nursing staff, these students are a valuable and irreplaceable resource; our results also show the unique issues that both hinder and help these students in their professional development; importantly, our results show the value of a joined‐up approach between universities and placement providers.

## 5 Strengths and Limitations

The study was conducted over four university sites in the UK, comprising a diverse sample, coupled with qualitative analysis methods yielding important insights into the effects of witnessing self‐harm and/or injury on MHN students. The study also adds new knowledge based on positive and negative experiences of MHN students, which could clarify how MHN students respond to witnessing self‐harm, support the service user and manage their own individual wellbeing. Limitations include uncertainties in the timings and severity of the events discussed by our participants, this resulted in the removal of information from the validated scale we used (Trauma Screening Questionnaire) as this was designed to be used 1 month after witnessing a traumatic event.

Many of our results are based on the qualitative data that was collected; however, the instrument used in data collection may have limited the depth of qualitative data that could be collected. Whilst participants provided detailed responses, future research would benefit from the usage of semi‐structured interviews to delve deeper into these issues.

## 6 Conclusions

This study has revealed a high proportion of MHN students witness self‐harm events and find the experiences traumatic; but if given appropriate support, they are better able to build resilience and positively react in the moment of the incident. These experiences can also assist in self‐management of ongoing emotions, as they are followed up with appropriate role modelling, support and guidance in how to manage the situation and beyond. Conversely, a lack of supervision/reflection and negative responses by registered and non‐registered staff can lead to distress and often unresolved issues. The majority of participants reported receiving training for responding to self‐harm events and managing subsequent emotions; however, some participants were critical of the level of preparation they received. This study did not find any data regarding the actual effectiveness of this training. Given the need to ensure student retention, and the likelihood of witnessing self‐harm, ensuring that MHN student preparation is effective should be an objective for universities and placement partners. Given that 50% of participants considered withdrawing from a course after witnessing self‐harm, there is a continuing need for rigorous research with a joined‐up approach between university and placement providers toward supporting MHN students to react, work through emotions and resolve issues. Therapeutic responses seem to help the MHN student develop their resilience. Overall, results suggest there are challenges present in the training and care of MHN students. However, we believe that overcoming these is an integral part of their journey, as this provides ways of building resilience. Rather than expect MHN students to pre‐emptively possess resilience, we instead encourage universities to focus on developing resilience in students prior to placements, a sentiment echoed by this project's participants.

A reality in the healthcare field is that the turnover rate of both current staff and students is likely to remain high, and current thinking suggests that the NHS will continue to deal with increased rates of self‐harm. Ultimately, if universities are to continue developing and training the next generation of nursing staff, then attention should be given to both developing core skills and students' holistic development.

## 7 Relevance to Clinical Practice

MHN students need better preparation for and support for working with people who self‐harm; this preparation needs to enable them to professionally and personally relate to the person in distress throughout their career. The involvement of practice partners is essential in preparing preregistration students toward becoming resilient future nurses; however, it is also important to highlight that some of our results suggest that current nursing culture, especially concerning the treatment of individuals with personality disorders, may alienate student nurses.

## Funding

Funding was received from Health Education England to undertake the study.

## Ethics Statement

Ethical permission to undertake the study was sought and granted from the University of Huddersfield School of Human and Health Sciences ‐ School Research Ethics and Integrity Committee (SREIC/2022/104) and confirmed by the other collaborating institutions.

## Conflicts of Interest

The authors declare no conflicts of interest.

## Data Availability

The data that support the findings of this study are available on request from the corresponding author. The data are not publicly available due to privacy or ethical restrictions.
